# Dr Robert George (Rob) Jones FRCPsych: Formerly Honorary Professor of Old Age Psychiatry, University of Nottingham

**DOI:** 10.1192/pb.bp.116.055632

**Published:** 2017-06

**Authors:** Tom Dening, Tom Arie

**Figure:**
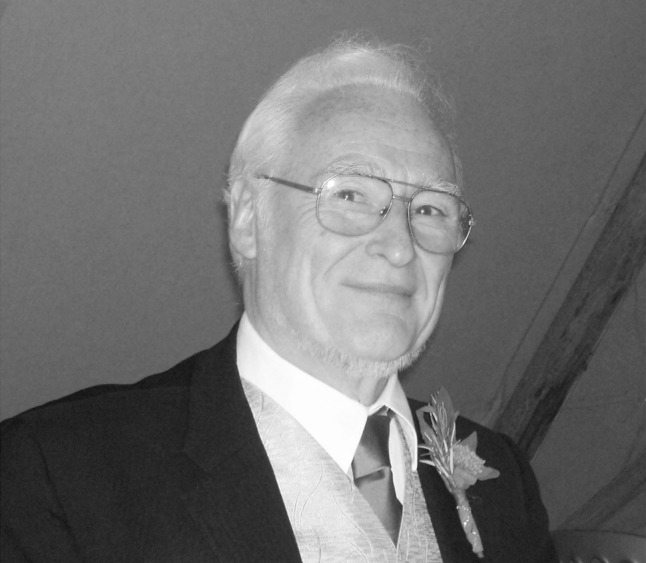


For over 30 years Rob Jones, who died recently at the age of 69, was a pillar of old age psychiatry in Nottingham and beyond. Qualifying in Manchester in 1970, he trained there in psychiatry and was particularly impressed with David Jolley's work in old age psychiatry. Following research with David Goldberg, he moved in 1980 to Nottingham as senior lecturer in psychiatry in the department of Health Care of the Elderly that Tom Arie had newly set up. This comprised physicians, psychiatrists and other health professions – Rob worked opposite John Bendall, the senior lecturer in medicine, and was a key figure in the ambitious 1-month combined attachment in old age medicine and psychiatry for all medical students. The novel joint department attracted wide interest, visitors and attached workers coming from home and abroad. Courses in psycho-geriatrics sponsored by the British Council or the World Health Organization brought workers from more than 30 countries to Nottingham and Nottingham staff were often invited abroad. Rob also contributed to a British Council course in Warsaw.

Rob's research included studies of outcomes for care home residents and of community provision; he also participated in major national studies and published widely. He was involved in collaborations that have shaped the practice of old age psychiatry across the UK and internationally. These included the DOMINO-AD clinical trial,^1^ which has shown that continuing anti-dementia drugs in people with moderate to severe Alzheimer's disease is worthwhile and does reduce the likelihood of entering a care home at least for a few months.

His most important research was around the care and services for older people with dementia, including, with Ian Rothera and others, a study of life expectancy after entering residential care.^2^ More recently, he provided the psychiatry input into the Medical Crises in Older People programme led by John Gladman. This documented just how many very elderly, frail and vulnerable people are admitted to hospital.^3^ His later work included studies of care for people with dementia living at home and at the time of his death he was involved in the NIHR Programme Grant PrAISED: Promoting Activity, Independence and Stability in Early Dementia, led by Rowan Harwood.

Rob was a busy clinician and led a district service for older people which served some of the most socially deprived parts of Nottingham, whenever possible taking services to people's own homes. He retired from clinical work in 2013 but continued to be a trustee of the local Radford Care Group, reflecting his passion for the well-being of people living at home, particularly those with dementia. He continued also to work for the university, heading the teaching programme in Health Care of the Elderly until his full retirement in October 2015. Of course, this was not the last we saw of him! He carried on with his research interests, as well as work on public involvement in dementia research – he organised the monthly Centre for Dementia seminars at the Institute of Mental Health. His contributions were recognised by the university with an honorary professorship.

Rob Jones was born in Paignton, Devon, into a family of proud Welsh ancestry. His father edited the local newspaper and this doubtlessly contributed to his insatiable lifelong interest in current affairs. At home he was permanently tuned into BBC Radio 4 and his colleagues were often deeply impressed by his detailed knowledge of world news and politics, to say nothing of lower league football (Torquay United in particular). In Manchester he met Diane and they married in 1971.

Rob was a man of warm and generous personality. He was regarded with respect and affection by all who knew him. His enthusiasm and commitment to the cause of older people was inspiring. For the last 20 years of his career he led academic old age psychiatry in Nottingham, as well as the Trent Dementia Research Network from 2004 until the establishment of the Nottingham Centre for Dementia in 2014. A particular mission was to keep us psychiatrists in close alliance with our colleagues in geriatric medicine, notably through the combined medical student programme. He was especially noted for bundles of papers in carrier bags, festoons of keys and lanyards at his neck, and his characteristic hearty laugh.

Notwithstanding his interest in people, he was quite private. For example, nobody he worked with was aware that Rob had been living with multiple sclerosis for 15 years. Indeed, he completed numerous half marathons during this time. He was definitely not one to complain. After he suffered a myocardial infarction in 2015, we were able to welcome him back to work. It is no surprise, therefore, that his sudden death on 23 May 2016 from a cerebellar haemorrhage came as a blow to all.

He leaves his wife Diane, and his children Haydn, Rhian, David and Siân, along with nine grandchildren.
